# Data-based clustering in prediction of cervical cancer DNA methylation using pan-cancer genetic and clinical data

**DOI:** 10.1093/bioadv/vbaf316

**Published:** 2025-12-14

**Authors:** Nidhi Pai, J Sunil Rao

**Affiliations:** Division of Biostatistics and Health Data Science, University of Minnesota, Minneapolis, MN 55414, United States; Division of Biostatistics and Health Data Science, University of Minnesota, Minneapolis, MN 55414, United States

## Abstract

**Motivation:**

Understanding the role of DNA methylation in oncogenesis, diagnosis, and treatment requires data sufficient in size and accuracy, but current epigenetic data is limited, especially for population groups underrepresented in research. We propose a framework for generating highly accurate DNA methylation predictions using classified mixed model prediction, incorporating a step to cluster patients into cross-cancer and cross-race groups.

**Results:**

Simulations show our framework more accurately predicts underlying mixed effects compared to regression prediction and naive estimates, extending previous work to the case where clusters are estimated from the data. We illustrate this framework using data from The Cancer Genome Atlas, uncovering clustering patterns and generating DNA methylation predictions for further analysis. Our work demonstrates how shared random effects can be leveraged to borrow strength across observations with similar methylation patterns.

**Availability and implementation:**

The methods are implemented in R and available at: https://github.com/nidhipai/dnam_cmmp.

## 1 Introduction

DNA methylation is an epigenetic modification that plays an important role in cancer. For example, hypermethylation of tumor suppressor genes can lead to reduced expression of those genes. In cervical cancer, specifically, this is associated with oncogenesis because it can lead to dysfunction of genes associated with apoptosis, the cell-cycle, the WNT-pathway, DNA repair, the FA-BRAC pathway, and mismatch repair, among other functions (Dueñas-González *et al.* 2005). Researchers have recently been interested in social epigenomics, which focuses on identifying social and environmental factors that influence human biology through the epigenome and how these factors contribute to health disparities (Mancilla *et al.* 2020). This is especially relevant to cervical cancer given that there are significant racial disparities in cervical cancer; for instance, the incidence rate among Black and American Indian/Alaska Native women are 68% and 19% higher, respectively, than that of White women (Cohen *et al.* 2023). Consider this illustrative example of the link between epigenomics and cancer disparities from Mancilla *et al.* (2020). Cervical cancer is strongly associated with HPV infection. A primary role of early protein 6 (E6), one of the most important oncogenic proteins of HPV, is to inhibit the function of tumor suppressor gene p53 via the ubiquitin pathway, an epigenetic modification (Dueñas-González *et al.* 2005). Benzoapyrene, a carcinogen found in cigarette smoke, increases the expression of E6, among other HPV oncogenes (Schmidt-Grimminger *et al.* 2011). American Indian women are known to smoke at much higher rates that White women (Arrazola *et al.* 2023); the connection between smoking and oncogene expression may be contributing to the observed disparities in cancer incidence. In such ways, epigenetic modifications serve as a link between the environment and the genome and may have significant implications on racial disparities in cancer. In addition, because DNA methylation is modifiable, it can be a therapeutic target (Lakshminarasimhan and Liang 2016). Furthermore, methylation patterns can also be used as diagnostic or prognostic biomarkers as well as a tool to differentiate patients into subgroups for tailored treatment (Lakshminarasimhan and Liang 2016). However, understanding the role of DNA methylation in oncogenesis, diagnosis, treatment, and personalized medicine, especially in a way that addresses and alleviates current health disparities, requires accurate data on many, diverse samples. Currently, epigenetic data is limited, with even less for racial minorities due to underrepresentation in research (Breeze *et al.* 2022). Given the importance of DNA methylation in cancer and this lack of data, the goal of this work is to generate highly accurate predictions of DNA methylation, especially for racial minorities, based on available genetic and clinical data.

Our strategy is to predict DNA methylation in samples from minorities by borrowing strength from other samples with similar patterns. We focus on cervical cancer; however, previous studies have pointed to cross-cancer similarities (Newton *et al.* 2017, Jiang *et al.* 2019), so we also borrow strength from samples of other cancers. Specifically, we cluster the methylation data into cross-race and cross-cancer groups with similar patterns and then fit mixed models with random effects by cluster. These models include clinical and genetic covariates but not environmental factors due to lack of data. As discussed above, environmental factors play an important role in DNA methylation, so any model without environmental factors is inherently misspecified. In a standard linear regression, this misspecification becomes part of the error. However, in our mixed effects framework, the cluster random effects absorb some of this error. Therefore, we can still predict the part of the environmental effects or other omitted covariates captured by the clusters, leading to a more robust model.

After the model is fit, predictions are made using the classified mixed model prediction (CMMP) methodology (Jiang *et al.* 2018). Given a new observation, CMMP makes predictions of the true mixed effect by identifying a group that the new observation belongs to and associating the corresponding random effect. This yields “smoothed” predictions of true DNA methylation that are less noisy and can be used for further analysis. Previous work has shown that the predictions from CMMP are more accurate than those generated by standard regression prediction (Jiang *et al.* 2018). Via simulation studies, we extend this result to show that these predictions more accurately recover the true underlying mixed effects compared with the noisy raw data. Furthermore, Jiang *et al.* (2018) assumed that the clusters were known a priori, and our simulations show that CMMP remains effective even when the number of clusters and cluster memberships are unknown. We then apply this approach to data from The Cancer Genome Atlas (TCGA) project, investigating patterns in clustering and assignment of random effects to test observations. Our work extends the applied analysis in Rao *et al.* (2021) by conducting an extensive simulation study and further investigating the predictions generated by CMMP.

The remainder of the paper is organized as follows. Section 2 describes the motivating data. Section 3 describes the clustering and mixed model methodology in more detail. Section 4 contains the results of the simulation study, and Section 5 contains the application and interpretation of results. We conclude with discussion in Section 6.

## 2 Data

### 2.1 Data description

Data from The Cancer Genome Atlas (TCGA) motivates the work and is used to illustrate the methodology. TCGA was a large cancer genomics program analyzing over 20 000 samples across 33 cancer types. The data is publicly available from cBioPortal (Cerami *et al.* 2012) and can be downloaded in R using the package TCGAretriever (Fantini 2024). For this study, we focus on patients with cervical and endocervical cancers (CESC), of which there are 295. As mentioned earlier, we will borrow strength from other patients to improve prediction accuracy, so we include 455 patients with lung adenocarcinoma (LUAD). LUAD was included based on prior results in Rao *et al.* (2021), who used a hybrid goodness-of-fit metric combining the gap statistic (Tibshirani *et al.* 2001) and Gini coefficient to identify optimal cancer types for joint modeling. See Section 1.3, available as supplementary data at *Bioinformatics Advances* online, for more discussion. Patients missing values for race, age, sex, or cancer stage were excluded, reducing the sample size to 254 cervical cancer patients and 401 lung cancer patients.

**Figure 1.**
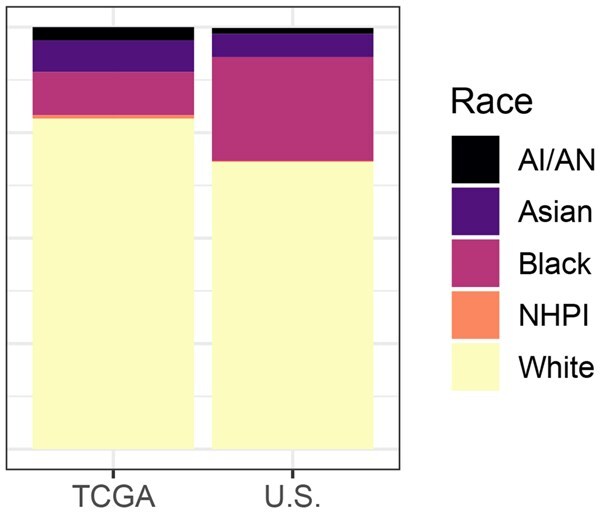
Racial composition of TCGA CESC patients and US CESC patients. In the TCGA data, Black patients are underrepresented. The racial composition of US patients was derived using incidence rates (Cohen *et al.* 2023) and population statistics (U.S. Census Bureau 2024).

Although TCGA is an extensive data source, it does not accurately reflect the racial composition of cervical cancer patients. Figure 1 compares the racial composition of the TCGA cervical cancer patients and US cervical cancer cases. Throughout the paper, we reference racial categories as they are in the TCGA data: American Indian or Alaska Native (AI/AN), Asian, Black or African American (Black), White, and Native Hawaiian or other Pacific Islander (NHPI). Of note, 24.6% of cervical cancer patients are Black, but after removing those with missing data, only 10.2% of the samples in the TCGA CESC data come from Black patients.

### 2.2 Data preprocessing

The raw CESC data contains 15 962 methylation markers (from the Infinium Human Methylation 450K BeadChip platform) and 24 245 gene features with somatic copy number alterations (CNA). Broadly, we excluded features with missing data and then further filtered variables to reduce their number and ensure low correlation between predictors. First, to prepare for an *M*-value transformation (see below), we treated any methylation values of 0 or 1 as missing. All variables that either contain missing data or are not shared between the CESC and LUAD datasets were excluded. We filtered the variables based only on the CESC data. Any methylation variables with standard deviation less than 0.2 were removed. For the CNA variables, the filtering was done in two stages. First, only CNA variables with a variance in the top 15% were kept. Second, for every pair of variables that had correlation higher than 0.7, one variable was eliminated such that the maximum amount of variables were retained. See Section 1.1, available as supplementary data at *Bioinformatics Advances* online, for additional discussion on the filtering of CNA variables. This process resulted in 2275 methylation variables and 65 CNA variables. Next, an *M*-value transformation $\left(y={\;\text{log}\;}_{2}\left(\frac{x}{1-x}\right)\right)$ was applied to each methylation feature to satisfy normality assumptions for the mixed model framework. To ensure that the pattern of methylation markers across cancers was observable, all methylation variables were standardized within the cancer type. In terms of clinical and demographic features, we focus on race, age, sex, and stage. For both cancers, stage variables were consolidated to Stage I, II, III, or IV.

## 3 Methods

### 3.1 k-means + CMMP framework

We use a mixed model framework in which participants are grouped into cross-cancer, cross-race clusters based on similarities in DNA methylation, and random effects are associated with these clusters. Specifically, first, the dataset is randomly split into a training and testing set, where all of the LUAD patients and 70% of the CESC patients are in the training set. To identify the aforementioned clusters, the k-means algorithm Lloyd (1982); MacQueen *et al.* (1967) is applied to the training set using all *M* methylation markers that pass preprocessing. The number of clusters *k* is chosen by calculating the gap statistic for 2 to 15 clusters and selecting the *k* best balancing a high gap statistic value but not too many clusters (see Tibshirani *et al.* 2001 for details). We chose k-means as the clustering algorithm for its simplicity and well-developed theoretical properties (e.g. Pollard 1981), but as shown in the Supplementary Materials, Section 1.2, available as supplementary data at *Bioinformatics Advances* online, CMMP is robust to this choice.

Next, let *i* refer to individuals and $j_{i}$ refer to *i’*s cluster. Note $j_{i}$ is fixed by subject and therefore does not change across methylation outcome. For the *m*th methylation outcome, m=1,…,M, we fit a mixed effects model


(1) $$y_{im}=x_{i}\beta_{m}+\alpha_{j_{i}m}+\epsilon_{im}=\theta_{im}+\epsilon_{im},$$

where $x_{ij}$ contains CNA variables, race, age, sex, stage, and cancer type (CESC or LUAD). We assume the $\alpha_{j_{i}m}∼N(0,G_{m})$ and $\epsilon_{im}∼N(0,R_{m})$ and that the $\alpha_{j_{i}m}$ and $\epsilon_{im}$ are independent.

We are interested in accurately predicting the mixed effect associated with new test observations. That is, we are interested in $\theta_{nm}=x_{n}\beta_{m}+\alpha_{Im}$ associated with $n_{\text{new}}$ test observations $y_{nm}=x_{n}\beta_{m}+\alpha_{Im}+\epsilon_{nm}$ indexed by the subscript *n* where $n=1,\ldots ,n_{\text{new}}$. In this paper, we do not know if any of the test observations are in the same group a priori, and so each test observation is assumed to be in its own group with $n_{\text{new}}=1$. Note that *I* is the unknown cluster assignment of the test observation. The random effect $\alpha_{Im}$ is assumed to come from the same distribution as the training set random effects. However, the test set data may or may not be from one of the training set groups. If so, then we can assign the appropriate training set random effect to the test set observations using the classified mixed model prediction (CMMP) (Jiang *et al.* 2018). If this matching assumption is not reasonable, then Jiang *et al.* (2018) also suggested an unmatched version of CMMP.

Specifically, we use test $\left\{y_{nm},x_{n}\right\}$, and the pretrained model to choose the best $\alpha_{Im}$ for test set observations. The prediction for $\theta_{nm}$ is then $x_{n}{\hat{\beta }}_{m}+{\hat{\alpha }}_{Im}$ in the matched version of CMMP. This is an atypical kind of prediction problem since we observe both $\{y_{nm}$, $x_{n}\}$. Thus, it is natural to consider predicting $\theta_{nm}$ with ${\bar{y}}_{nm}$ directly. However, the $y_{nm}$ contains error $\epsilon_{nm}$, and so one may be able to do better by using CMMP from the training data model and borrowing strength across clusters through the model estimates. This is especially relevant when we have a small number of test observations known to be from the same group, and here, each observation is in its own group a priori. Jiang *et al.* (2018) showed that under the matched or unmatched assumption for the test data, that predictions for the test data mixed effects were consistent. However, that was under the assumption that the clustering was known a priori. Exploring the behavior of CMMP when the clustering is estimated from the data is a key development of this paper.

A close inspection of Equation (1) reveals that the model decomposes methylation into a piece explained by covariates and a piece explained by the cluster effects. Because these clusters are cross-race and cross-cancer, predictions for a single observation borrow strength from other races and cancers. This is not possible in standard linear regression. In Section 4, we show via simulations that the predictions generated using the k-means and CMMP approach (k-means + CMMP) are usually more accurate than linear regression (“regression prediction”) or using $y_{nm}$ alone (the “naive estimate”). For real data, Algorithm 1 provides a method of calculating the MSE of mixed effect predictions via parametric bootstrap for one methylation outcome, and results in Section 5 show that k-means + CMMP estimates have lower MSE than those of competing methods.

**Algorithm 1** Parametric bootstrap MSE calculation. **Input:**  ${\hat{\beta }}_{m},{\hat{G}}_{m},{\hat{R}}_{m}$ from fitting mixed model 1 and ${\hat{I}}_{m}$ from CMMP

**Output:** MSE of ${\hat{\theta }}_{nm}$

For b=1,…,B:

1. Generate *k* random effects $\alpha_{j_{i}mb}$ from $N(0,{\hat{G}}_{m})$.

2. For the training data, calculate $y_{\mathrm{imb}}=x_{i}{\hat{\beta }}_{m}+\alpha_{j_{i}mb}+\epsilon_{\mathrm{imb}}$ where $\epsilon_{i}\;∼N(0,{\hat{R}}_{m})$.

3. For observation *n* in test data, let ${\hat{I}}_{m}$ be its cluster and generate $y_{\mathrm{imb}}=x_{i}{\hat{\beta }}_{m}+\alpha_{{\hat{I}}_{m}mb}+\epsilon_{\mathrm{imb}}$ where $\epsilon_{i}∼N(0,{\hat{R}}_{m})$.

4. Use CMMP to fit a mixed model on the training data $y_{\mathrm{imb}}$ and obtain mixed effects predictions ${\hat{\theta }}_{\mathrm{nmb}}$ for the test data.

The MSE of ${\hat{\theta }}_{nm}$ is estimated by $\frac{1}{B}\sum_{b=1,\ldots ,B}{\left({\hat{\theta }}_{\mathrm{nmb}}-{\hat{\theta }}_{nm}\right)}^{2}$.

### 3.2 Software

All analyses were performed using R version 4.3.0 (R Core Team 2023). The gap statistic is implemented in the R package cluster (Maechler *et al.* 2022). Code to replicate both the real data analysis and the simulation study can be found in the following GitHub repository: https://github.com/nidhipai/dnam_cmmp.

## 4 Simulation study

### 4.1 Simulation setup

Because the prediction target $\theta_{im}$ is not directly observable, the predictive performance of this method is best studied via simulation. Data is generated according to the following steps:

1. Independently for each covariate (65 CNA variables, cancer type, stage, age, sex), generate n=655 samples from the empirical distribution of the covariate. For sex, the empirical distribution is specific to the cancer type, i.e., all rows with cancer type CESC are assigned female sex. Because we are interested in the effect of under-representation of racial minorities on predictive accuracy, we introduce a simulation parameter *b* for “bias”, which is the proportion of participants that are White. After sampling b×n rows to be assigned White, the remaining rows are assigned a race by sampling from the empirical distribution of race after removing the White category.
2. The errors $\epsilon_{im}$ are independent with mean 0 and variance .9, which reflects the estimated error variances in the real data. For each methylation outcome variable *m*, where m=1,…,2275, the fixed effect $\beta_{m}$ is generated from a normal distribution with mean 0 and variance .03, chosen such that the signal-to-noise ratio $\mathrm{SNR}=\frac{\text{Var}(X\hat{\beta })}{{\hat{\sigma }}^{2}}$ is close to the estimated signal-to-noise ratios in the real data.
3. Each observation *i* is randomly assigned a cluster $j_{i}$ from 1 to *c*, where the number of clusters *c* is simulation parameter. The random intercepts $\alpha_{jm}$ are generated from a 2275 dimension multivariate normal distribution with a “clumpy dependence” relationship. More specifically, the variance of the random intercepts $\alpha_{jm}$ is $\sigma^{2}$, which is a simulation parameter. Then, two blocks of 2275/4 methylation variables each are chosen and every pair of outcomes in the same block has covariance .07. The remaining outcomes are uncorrelated.

In short, simulation parameters in data generation are the sampling bias (b=.5,.6,.681 (unbiased), 0.78 (TCGA bias), 0.9), the number of clusters (c=1,2,4,6,8,10,16), and the variance of the random intercepts (σ=.1,.2,.5,1,2). In addition, the k-means + CMMP method requires specifying the number of clusters *k*, which we vary (k=1,2,4,6,8,10,16,20) in the simulation study. Because testing each combination of parameters is too computationally burdensome, we fix a default set of parameters that reflects the real data (b=.78, c=6, σ=.2, k=6) and vary one parameter at a time. 300 datasets are analyzed for each simulation configuration. For each simulated dataset, we compare (i) the k-means + CMMP method, (ii) regression prediction, that is, fitting a linear regression model with CNA variables, race, age, sex, stage, and cancer type as predictor variables, (iii) the naive estimate $y_{nm}$, and (iv) oracle CMMP, which uses the true clusters instead of estimating the clusters with k-means. Oracle CMMP provides an upper bound on the performance of the k-means + CMMP method and is only possible in simulations. To compare the methods, we calculate mean squared error (MSE) between the estimated mixed effect ${\hat{\theta }}_{nm}$ and the true mixed effect in the test set. We also calculate the MSE restricted to racial minorities to study the effect of under-representation.

### 4.2 Simulation results

**Figure 2.**
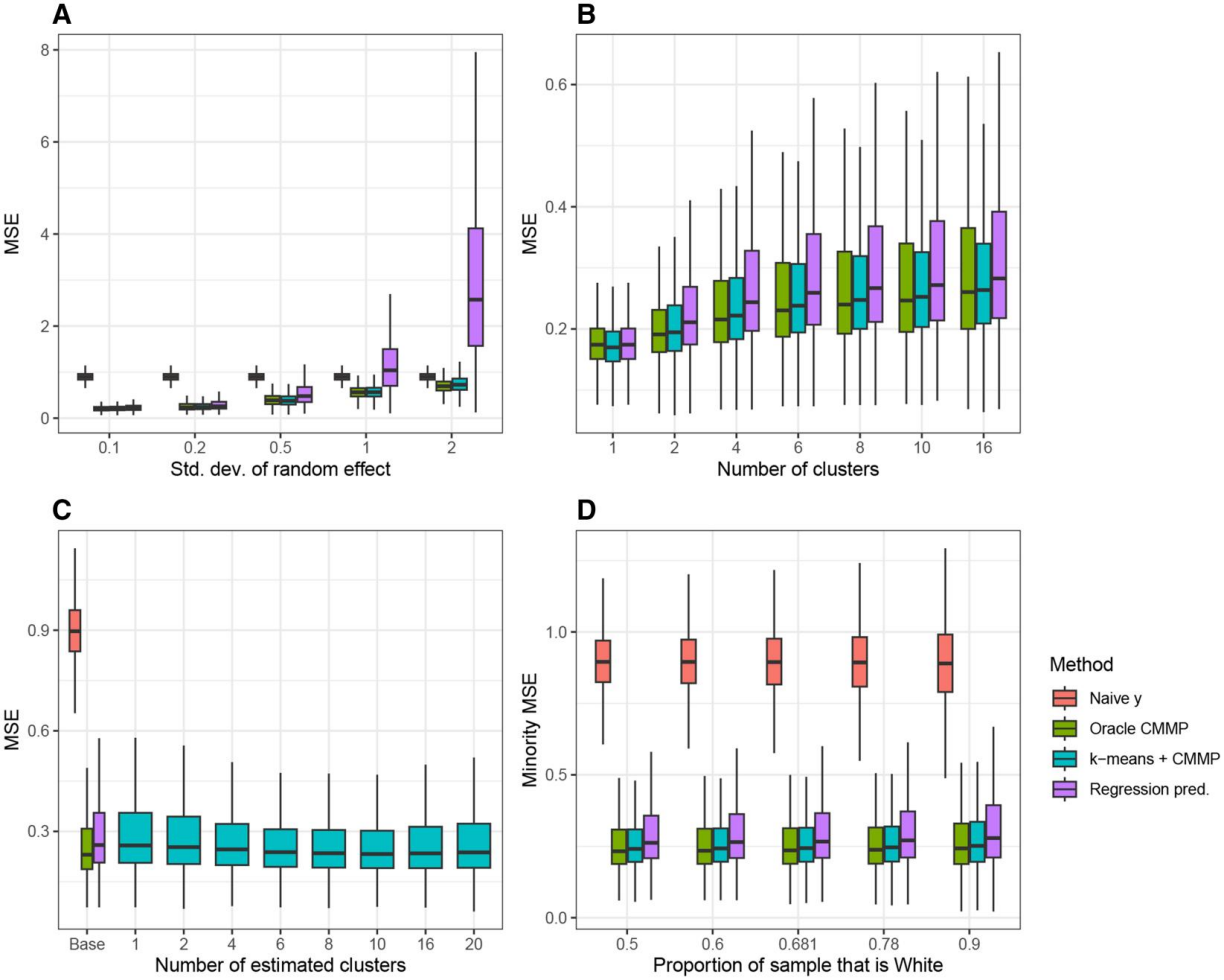
Distribution of MSE in predicting true mixed effect varying different simulation parameters. (A) MSE distributions across 300 simulated datasets for different standard deviations of the random effect. The four methods compared are (i) the naive estimator, $y_{nm}$, (ii) oracle CMMP, which is given the true clustering, (iii) our framework k-means + CMMP, and (iv) linear regression prediction. (B) MSE by number of clusters in the data; the naive estimator is omitted as its performance is centered at 0.9. (C) MSE by *k*, the number of clusters specified in k-means + CMMP; the other methods are only shown once since choosing *k* is not required. (D) MSE calculated from only patients in racial minorities.

For each value of each simulation parameter, we plot a boxplot of MSE across simulation trials and outcomes in Fig. 2. In general, across all simulations, the MSE for the naive estimator $y_{nm}$ is centered at 0.9, the variance of $\epsilon_{im}$; this is because the squared error here is ${\left[y_{nm}-\theta_{nm}\right]}^{2}=\epsilon_{nm}^{2}$, so the expected squared error is $E\epsilon_{nm}^{2}=.9$. Also, the performance of k-means + CMMP is approximately lower-bounded by the performance of oracle CMMP, since the oracle CMMP method is using the true clustering.

**Figure 3.**
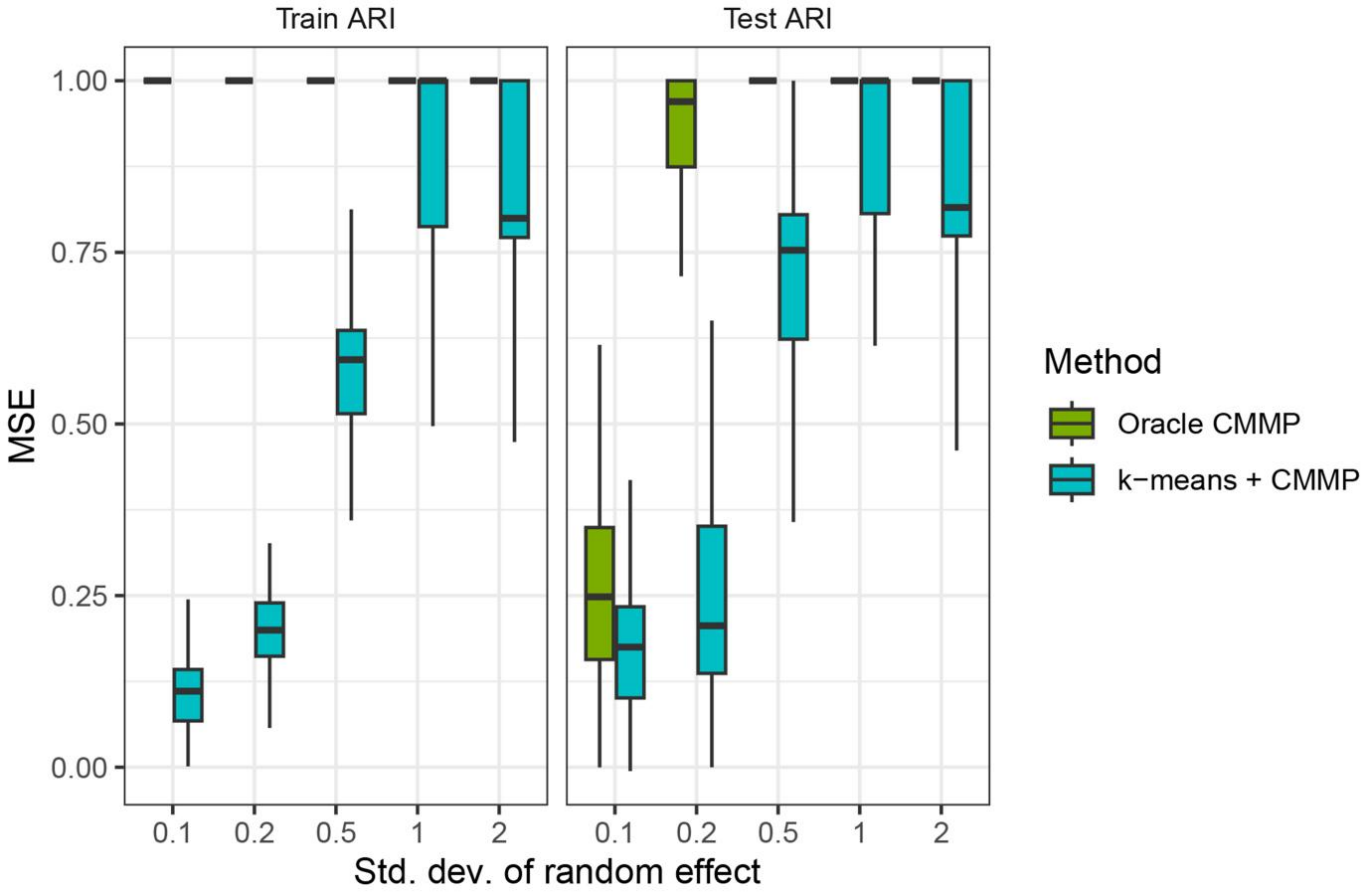
Similarity of clustering in train and test sets to data-generating clusters for CMMP methods. Similarity is measured using adjusted rand index (ARI). For the training data, oracle CMMP is given the true clustering, so the train ARI is 1, whereas for k-means + CMMP, the ARI is determined by k-means. For both methods, the test ARI is determined by how CMMP matches random effects to test observations. Given a test observation, CMMP is run on each methylation outcome separately; the observation’s cluster assignment is the most common cluster assignment among outcomes.

Figure 2A shows distribution of MSE across outcomes for different values of σ, the standard deviation of the random intercept. At the default value of 0.2, k-means + CMMP and oracle CMMP are slightly outperforming regression prediction and largely outperforming the naive estimator. As $\sigma^{2}$ increases, the random effect contributes more to the mixed effect, and regression prediction performs relatively poorly, as it is unable to capture the random effect contribution. Furthermore, the performance of the two CMMP methods also decreases as $\sigma^{2}$ increases; as $\sigma^{2}$ increases, the overall variance of $y_{im}$ increases as well. For all values of $\sigma^{2}$, the performance of k-means + CMMP is close to that of oracle CMMP. To understand this more, Fig. 3 shows the clustering accuracy for the train and test sets, measured with Adjusted Rand Index (ARI) (Hubert and Arabie 1985), which quantifies the similarity between two data groupings and is upper-bounded at 1. Because oracle CMMP is given the true clustering of the training data, the training ARI is 1 for all simulations, whereas the train ARI for k-means + CMMP is determined by k-means. The test ARI for both methods is determined by how CMMP matches random effects to test observations. For a given test observation, CMMP is applied to each outcome separately, so we define the cluster assignment of the observation as the most common cluster assignment among outcomes. As $\sigma^{2}$ increases and the cluster random effect contributes more, the train ARI for k-means + CMMP increases. Similarly, the test ARI also increases for both methods, and except for at σ=0.2, the distribution of test ARI for k-means + CMMP is somewhat lower but not very different from that of oracle CMMP. The similar performance of the two CMMP methods in Fig. 2A may be because the test set clustering is similarly accurate compared to the true clusters.

Next, Fig. 2B compares MSE across different values of *c*, the true number of clusters. Across all values of *c*, the three other methods all outperform the naive estimator, so it is omitted from plots. As *c* increases, all three methods perform worse, although the median MSE for k-means + CMMP remains under that of regression prediction. Figure 2C shows the MSE when choosing different values of the number of estimated clusters *k* in k-means + CMMP; there are c=6 clusters in data generation for all simulations in this set. Notably, the performance of k-means + CMMP does not fall even when significantly overestimating the number of clusters. In general, choosing the hyperparameter *k* is the least straightforward aspect of k-means, but the k-means + CMMP method is quite robust to the value of *k*. Finally, Fig. 2D shows the distribution of MSE as well as MSE calculated only from patients of racial minorities across degrees of underrepresentation. Predictably, as the proportion of the sample that is White increases, the minority MSE tends to increase as well, as there are fewer observations from patients who are not White.

In summary, using simulated data similar to the TCGA data, the k-means + CMMP method predicts the true underlying mixed effect more accurately than regression prediction or the naive estimator in most cases. Furthermore, the method is robust to *k*, the number of clusters specified.

## 5 Application

### 5.1 Clustering

**Figure 4.**
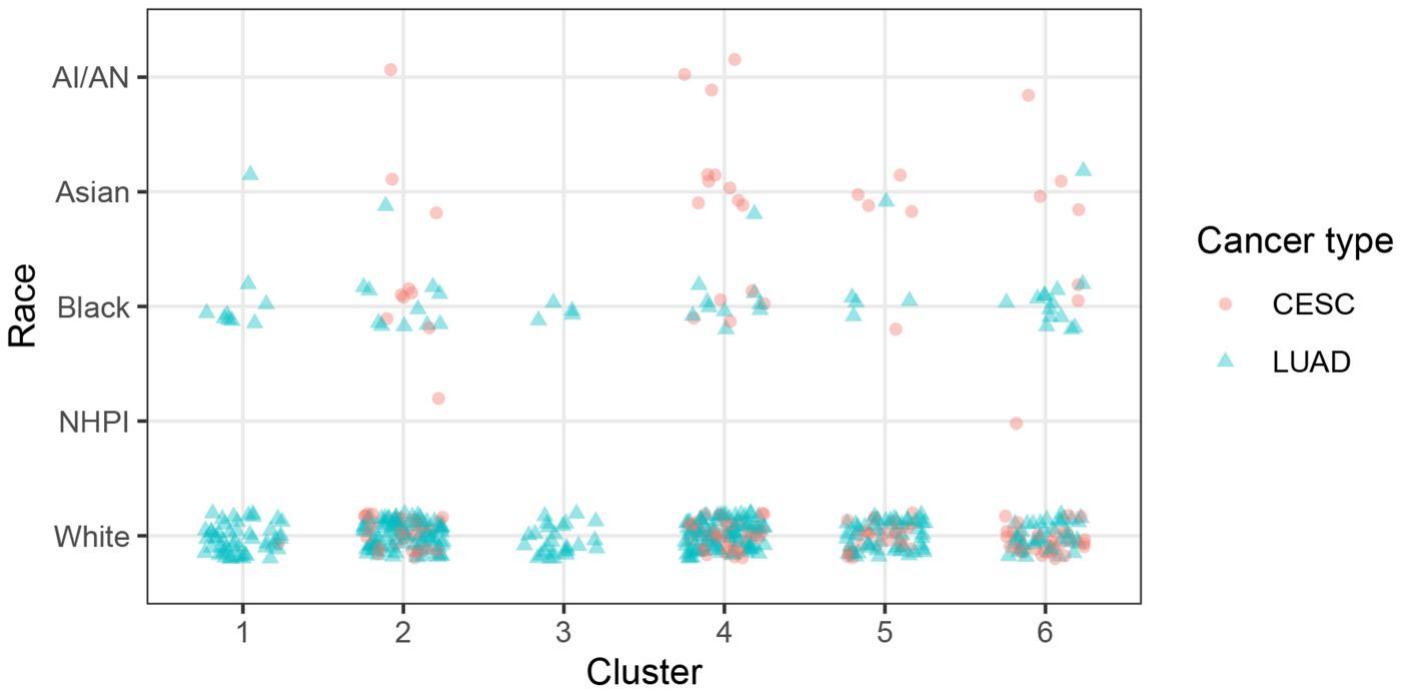
Cluster composition by cancer type and racial group. Only those in the training set are included in the k-means clustering. Clusters contain patients from both cancer types and multiple races, except clusters 1 and 3 which contain only LUAD patients.

Using the gap statistic Tibshirani *et al.* (2001) for selecting the optimal number of clusters, the methylation values of patients in the training dataset are grouped into k=6 clusters using the k-means algorithm. Figure 4 shows the composition of each cluster by race and cancer type. Clusters are cross-race and cross-cancer type; that is, most clusters contain patients from a mixture of races and cancer types.

**Figure 5.**
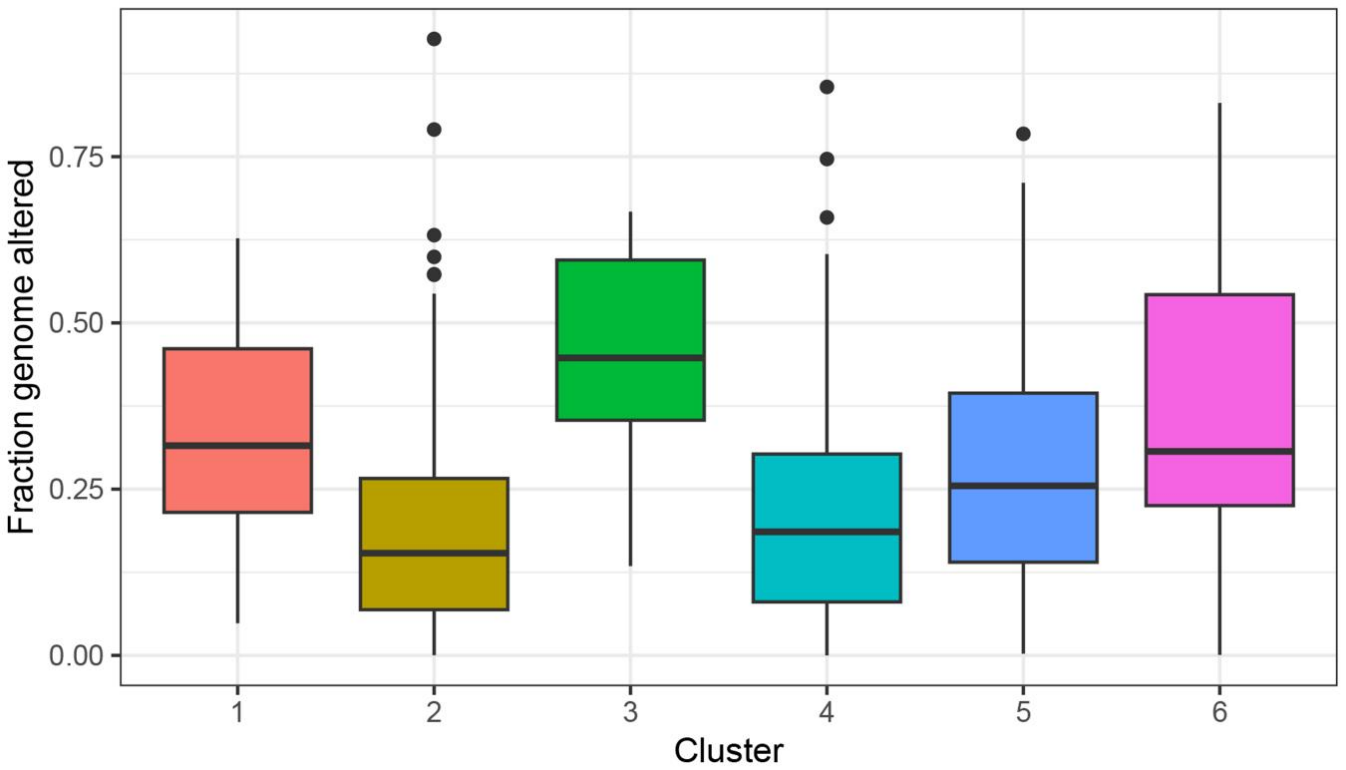
Fraction of genome altered by cluster. Only observations in the training set are clustered in k-means. Clusters are significantly associated (adj. P<.001) with fraction genome altered, the proportion of the genome affected by copy number alterations. Cluster 3, which consists exclusively of LUAD patients, has a much higher median fraction genome altered than clusters 2 or 4.

Next, to better understand the drivers of clustering, we looked for associations between cluster and covariates using an ANOVA *F*-test and a chi-squared test for continuous and categorical covariates, respectively; a Bonferroni correction was used to adjust *p*-values for multiple testing. Clusters are significantly associated with age (adj. P=.008), fraction of genome altered (adj. P<.001), sex (adj. P=.008), and cancer type (adj P<.001). Clusters were not significantly associated with race or cancer stage. For each cluster, Fig. 5 shows the distribution of fraction of genome altered, which is the proportion of the genome affected by copy number alterations. The median fraction genome altered varies between the clusters; for example, cluster 3, which consists exclusively of LUAD patients, has a much higher median than clusters 2 or 4.

### 5.2 Modeling

**Figure 6.**
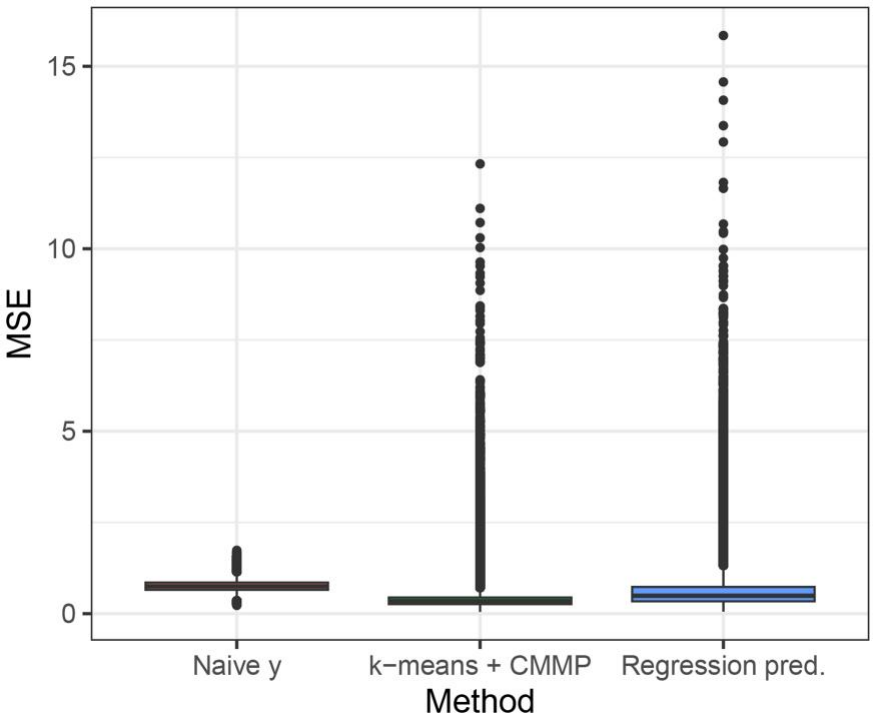
Distribution of MSE in predicting $y_{nm}$ of test set observations across outcomes using $y_{nm}$ naively, k-means + CMMP, and regression prediction. The estimates are calculated via parametric bootstrap as described in Algorithm 1.

For the real data, we compare k-means + CMMP to (i) regression prediction and (ii) using $y_{nm}$ naively by calculating the MSE of ${\hat{\theta }}_{nm}$ according to Algorithm 1. This error measurement is a proxy because the prediction target, $\theta_{nm}$, is unobservable. Because $\theta_{nm}$ is known in simulations, Section 4 more accurately illustrates the performance of each method under different scenarios. Note also that because the MSPE is evaluated on a test set, fixing the number of clusters based on the training set does not bias the results. With this considerations in mind, Fig. 6 shows the relative performance of k-means + CMMP in the real data; each boxplot shows the MSE in predicting $y_{nm}$ of observations, across test observations and across methylation outcomes. Overall, the median MSE for k-means + CMMP is lower than that of the other two methods.

Next, to understand the relative contributions of the clinical and demographic variables (race, sex, age, stage, and cancer type) vs. the genetic variables to the fixed effects in the model, we calculate partial $R^{2}$ values using the *R* package partR2 (Stoffel *et al.* 2021). Figure 4, available as supplementary data at *Bioinformatics Advances* online, shows the distributions of $R^{2}$ values over all the methylation marker models. The first and second panels show the partial $R^{2}$ values for the clinical/demographic variables and the genetic variables, respectively. The third panel shows the full model $R^{2}$. It is evident that the genetic variables explain more variability in the outcome than the clinical/demographic variables, and the genetic variables are responsible for most of the variability explained by the model.

The random effects can compensate for the variation that is not captured by any of the fixed effects because they adjust the predictions from the fixed effects. Thus the magnitude of random effects provide a signal for how well the fixed effects explain the response; a large magnitude of the random effect indicates that the fixed effects are contributing relatively less. To understand the contribution of the random effects in this case, Fig. 5, available as supplementary data at *Bioinformatics Advances* online, plots the distribution of test set random effects by cluster over all methylation outcomes. Recall that for each methylation outcome, each observation in the test set is assigned the random effect of the best-matching training set cluster. The distribution of assigned random effects is quite different by cluster. For example, for cluster 6, the random effects are centered around −0.5 and the distribution is relatively tight, indicating that the fixed effects are underestimating the methylation for the vast majority of the methylation markers. On the other hand, for clusters 1 and 3, the distribution is bimodal with a larger variance, so the random effects are contributing in different ways to different methylation outcomes. In this way, the random effects illustrate the limitations of the model fixed effects for different patients and methylation outcomes.

## 6 Discussion

In summary, we have developed a novel mixed model framework for accurately predicting DNA methylation from clinical and genetic variables by extending CMMP to the case where clustering is not known a priori. Via simulations, we showed that this k-means + CMMP method can outperform naive estimates and regression prediction in predicting a mixed effect, even when the clustering is data-driven, and the results are robust to *k*, the number of clusters. This methodology was applied to the TCGA CESC data, where we focused on improving predictions for underrepresented groups by leveraging data from similar patients across cancers and racial groups.

In this paper, we used a “bundle of sticks” approach to race (Sen and Wasow 2016). More specifically, we are not viewing race as an immutable characteristic which causes specific patterns in DNA methylation. Rather, we are considering racial categories as collections of factors such as region of ancestry, diet, treatment by institutions, physical characteristics, etc. The race variable captures some of these elements to improve predictive accuracy. It is reasonable to utilize our framework to improve predictions for any other type of population groups affected by underrepresentation in research and health disparities.

There are several other avenues of future research. First, in this analysis, we assumed that $y_{nm}$ was observed for both the training and test observations and focused on predicting the unobserved mixed effect $\theta_{nm}$. Jiang et al. (2018) also extend CMMP to predicting future observations using covariates only, but this requires repeated observations known to be from the same cluster. Second, we fit a different mixed model for each methylation outcome, but one could fit a multivariate model and use a multivariate extension of CMMP for prediction. This work is underway (Zhang *et al.* 2025). Third, this paper studied the behavior of k-means + CMMP via simulation, but it would also be useful to extend theoretical work on CMMP to the case where clusters are estimated with k-means. Fourth, it may also be possible to devise a nonparametric Bayesian approach similar to the methods above, which could help relax the normality assumption in the mixed models. Finally, our approach could be extended to incorporate additional -omics data types, either as covariates to improve prediction, or as new outcomes.

An important limitation of this work is the small number of Black CESC patients, reducing the strength of subgroup-specific conclusions. This reflects a broader lack of representation in public genomic databases, as discussed in the introduction. Because DNA methylation plays a critical role in understanding and treating cervical cancer, our goal is to improve prediction accuracy of DNA methylation within the constraints of the available data. While methodological advances can partially mitigate such imbalances, truly equitable progress ultimately requires more representative data collection.

## Supplementary Material

vbaf316_Supplementary_Data

## Data Availability

The data underlying this article are available in the Genomic Data Commons, https://portal.gdc.cancer.gov/.
