# Supplementary material for: Data-based clustering in prediction of cervical cancer DNA methylation using pan-cancer genetic and clinical data
Source: Bioinform Adv. 2025 Dec 14;6(1):vbaf316. doi: 10.1093/bioadv/vbaf316 (PMC12776347; doi:10.1093/bioadv/vbaf316)
Supplement: vbaf316_Supplementary_Data [file vbaf316_supplementary_data.pdf]

Supplementary Materials for
Data-based clustering in prediction of cervical cancer DNA methylation using pan-cancer genetic and clinical data

by

Nidhi Pai, J. Sunil Rao

S1 Sensitivity analyses

S1.1 Filtering of CNA variables

The dataset initially contains 24,247 CNA variables, which are highly collinear. To reduce dimensionality, we applied a two step-filtering process. First, we removed any CNA variables with a variance below of the top 15%. Second, for every pair of variables with a correlation higher than .7, one variable was eliminated such that the maximum amount of variables were retained. These steps are similar as those in [4], except in the first step, they only kept variables with a variance in the top .5%. We chose a higher cutoff to be more conservative and avoid filtering out potentially important variables.

To evaluate the sensitivity of our results to this choice, we reran the analysis using two alternative cutoffs, 10% and 20%. Table S1 summarizes the number of variables filtered and retained at each step. As expected, increasing the cutoff and retaining more variables after the first filtering step leads to a larger number of correlated variables being removed in the second step, but still results a slightly higher number of final covariates overall.

Variance percentile cutoff	Filtered at step 1	Remaining after step 1	Filtered at step 2	Remaining after step 2
10%	21822	2425	2379	46
15%	20591	3656	3591	65
20%	19386	4861	4782	79

Table S1: Number of CNA variables filtered and retained after each of the two filtering steps, under different variance cutoffs for the first step.

Figure S1 compares the distribution of MSE of DNA methylation estimates across methylation markers for the three variance cutoffs and regression prediction for reference. The boxplots are analogous to Figure 6 in the main paper, but the error is not calculated with parametric bootstrap in Algorithm 1 due to computational

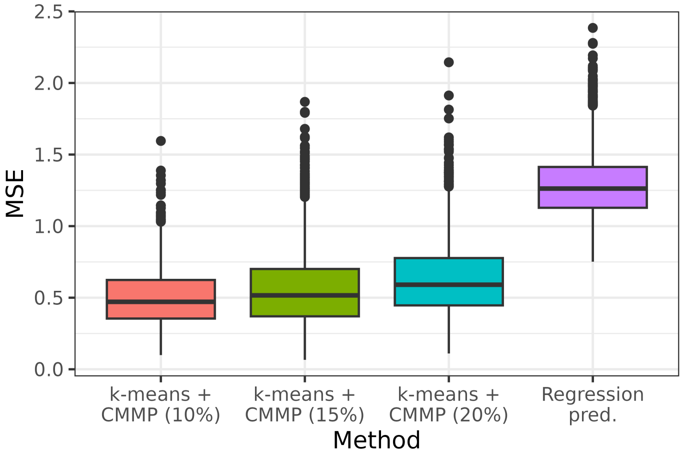

Figure S1: Distribution of prediction error over DNA methylation markers for three variance cutoffs and regression prediction.

constraints. Instead, following [1], the MSE is calculated as $(\hat{\theta}_{nm} - y_{nm})^2$; that is, the observed data is treated as the truth. While this metric is biased for the true estimates, relative differences between methods are preserved. As shown in Figure S1, CMMP predictions, using any of the three cutoffs, outperform regression prediction. As the variance cutoff increases and more variables are included in the model, the prediction error increases, likely due to overfitting from a larger number of covariates. This effect could potentially be mitigated by using a high-dimensional extension of CMMP, as proposed by [2].

S1.2 Clustering method

In the simulations and application, the clustering step is performed with k-means. [4] considered k-means, hierarchical clustering, affinity propagation, and partitioning around medoids (PAM) for the clustering step, finding that the methods performed similarly. We selected k-means for its simplicity and well-developed theoretical properties (e.g., [3]), expecting that the results would be relatively robust to the specific clustering algorithm chosen.

As a sensitivity analysis, we compare k-means to hierarchical clustering and Gaussian mixture models, fixing the number of clusters at 6 for comparability to the original analysis. As shown in Figure S2, the MSE of DNA methylation estimates from the three methods are similar and consistently outperform regression prediction. The differences between clustering methods may be due to the suitability of each method to this dataset or the lack of individualized hyperparameter tuning for each method, i.e., fixing the number of clusters at 6. (Note that the MSE calculation in Figure S2 is as described in Section S1.1 for Figure S1, not the parametric bootstrap in Algorithm 1.) Overall, these findings combined with the analysis in [4] suggest that the CMMP framework is not highly sensitive to the choice of clustering algorithm, but there may be

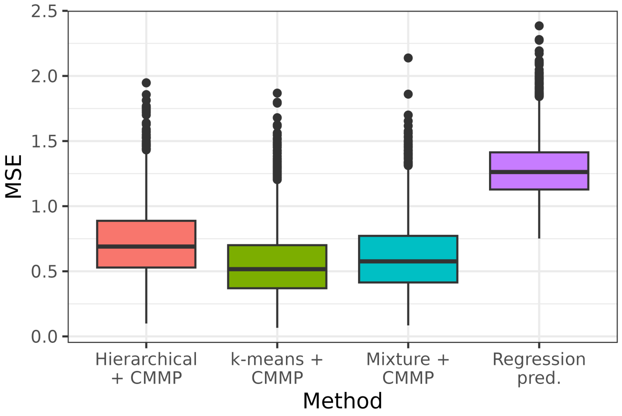

Figure S2: Distribution of prediction error over DNA methylation markers for three clustering methods (hierarchical clustering, k-means, and mixture models) and regression prediction.

other contexts where results from different clustering methods diverse more substantially.

S1.3 Cancer types included

We selected LUAD as the additional cancer type based on the results presented in [4]. In that study, the authors proposed a hybrid goodness of fit metric that combines the gap statistic and the Gini coefficient to identify suitable cancer types for joint modeling. In addition to CESC and LUAD, the authors also considered breast cancer (BRCA), colon adenocarcinoma (COAD), and kidney renal clear cell carcinoma (KIRC). Out of the cancer types available in TCGA, these four were considered because they (1) were either female-specific or not constrained by sex, (2) included race information and at least some Black patients, and (3) contained methylation data. Among these, the combination of CESC and LUAD yielded the highest value of the hybrid goodness of fit metric. Adding a third cancer type did not substantially improve the metric, so only two cancers were used for parsimony.

Here, we reexamine the selection of cancer types. Because the TCGA2STAT package used in [4] has been deprecated, we instead obtained TCGA data though TCGAretriever. The COAD dataset available through TCGAretriever did not include methylation data and therefore could not be evaluated. We replicated the analysis of [4], calculating the hybrid goodness of fit metric across the available combinations of cancer types. Specifically, for each combination of cancer types and between $k = 2$ and $k = 15$ clusters, we computed the gap statistic $\text{Gap}(k)$ and selected the optimal number of clusters k^* following [5]. Next, we calculated the Gini coefficient quantifying the heterogeneity of cluster composition with respect to cancer types

$$\text{Gini}(k^*) = \max_{i \in 1,\ldots,k^*} \sum_{j=1}^{C} \frac{n_{ij}}{n_i} \left(1 - \frac{n_{ij}}{n_i}\right),$$

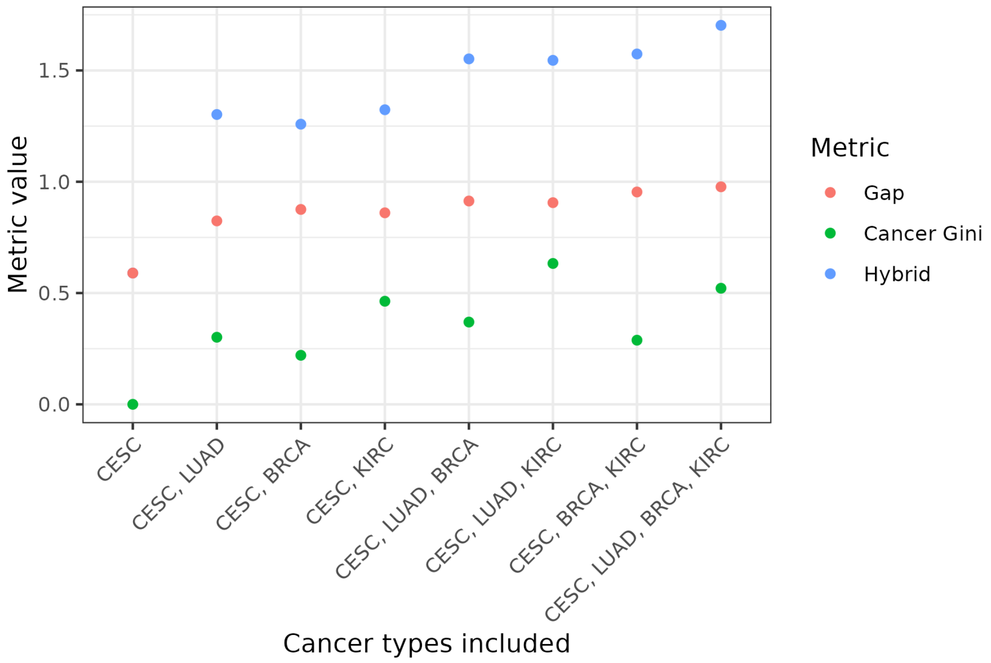

Figure S3: Hybrid goodness of fit metric across cancer type combinations.

where n_i is the number of patients in cluster i, C is the number of cancer types being considered, and n_{ij} is the number of patients in cluster i with cancer type j. The hybrid metric for the cancer type combination is then $\text{Gap}(k^*) + \text{Gini}(k^*)$. The results in Figure S3 show the metric for each combination of cancer types. These findings differ from those reported in [4], likely due to differences in the underlying datasets and preprocessing methods. Here, among the combinations of two cancer types, the CESC and KIRC combination has a higher hybrid score than CESC and LUAD, but only by about .021. For comparability to [4], we keep CESC and LUAD as the cancer types in the main simulations and application.

S2 Supplementary Figures

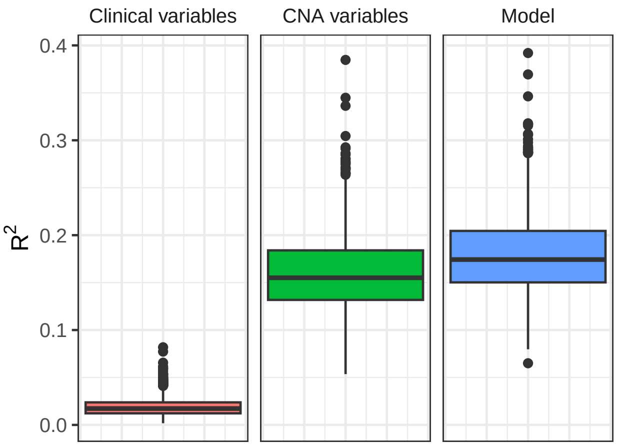

Figure S4: Distributions of partial R^2 values by model, one model for each methylation variable. The first and second panels show the partial R^2 values for the clinical/demographic variables (race, sex, age, stage, and cancer type) and the genetic variables, respectively. The third panel shows the full model R^2. The genetic variables explain more variability in the outcome than the clinical/demographic variables.

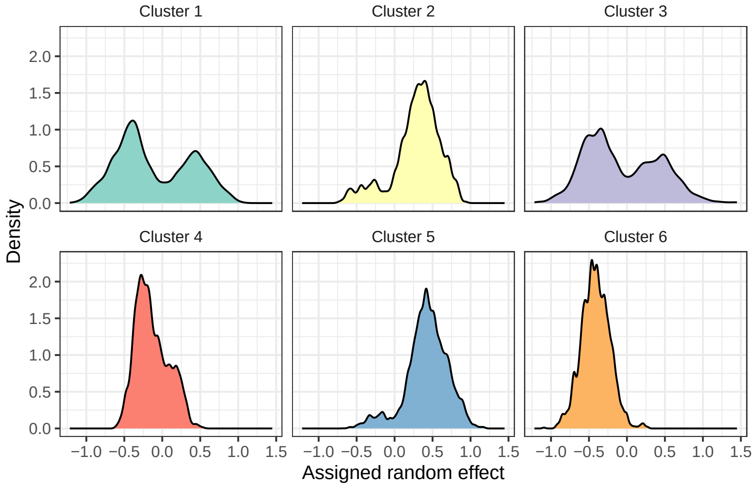

Figure S5: Distribution of test set random effects by cluster over all methylation outcomes. For each methylation outcome, each observation in the test set is assigned the random effect of the best-matching training set cluster.

References

[1] Jiming Jiang, J Sunil Rao, Jie Fan, and Thuan Nguyen. Classified mixed model prediction. *J. Am. Stat. Assoc.*, 113(521):269–279, 2018.

[2] Mengying Li. *New Methods on Classified Mixed Model Prediction: Theory and Application.* PhD thesis, University of Miami, 2020.

[3] David Pollard. Strong consistency of k-means clustering. *The Annals of Statistics*, pages 135–140, 1981.

[4] J Sunil Rao, Hang Zhang, Erin Kobetz, Melinda C Aldrich, and Douglas Conway. Predicting DNA methylation from genetic data lacking racial diversity using shared classified random effects. *Genomics*, 113(1):1018–1028, 2021.

[5] Robert Tibshirani, Guenther Walther, and Trevor Hastie. Estimating the number of clusters in a data set via the gap statistic. *J. R. Stat. Soc. B*, 63(2):411–423, 2001.
